# Failure of timely removal of central and peripheral venous catheters after antibiotic therapy in nursing homes

**DOI:** 10.1017/ice.2025.17

**Published:** 2025-05

**Authors:** Amarah Mauricio, Joshua B. Hsi, Tom Tjoa, Raveena D. Singh, Shereen Nourollahi, Raheeb Saavedra, Bardia Bahadori, Mohamad N. Alsharif, Steven Tam, Justin Chang, Syma Rashid, Shruti K. Gohil

**Affiliations:** 1 Division of Infectious Diseases, University of California Irvine, School of Medicine, Irvine, CA, USA; 2 Division of Geriatric Medicine and Gerontology, University of California Irvine, School of Medicine, Irvine, CA, USA; 3 Epidemiology & Infection Prevention, UC Irvine Health, Orange, CA, USA

## Abstract

Each day a venous catheter is retained poses unnecessary safety risks. In a retrospective evaluation of central/peripheral lines in nursing home residents receiving antibiotics, 80% were retained beyond antibiotic treatment end and nearly one third were retained longer than a week. Interventions for timely catheter removal are urgently needed.

## Background

Each year in the United States, 2.7 million peripherally inserted central catheters (PICCs) are placed,^1^ a large proportion of which are managed postdischarge for antibiotic therapy. Continuation of intravenous antibiotic therapy is a common reason for nursing home placement.^2^ Completion of antibiotic therapy should signal line removal and ideally occur on the same day therapy is concluded in a nursing home. We sought to evaluate the extent to which central venous catheters that had exceeded their designated usage were promptly removed in nursing homes.

## Methods

We conducted a retrospective analysis of the SAFER Lines nursing home study.^3^ In brief, this study involved an evaluation of central and peripheral catheter insertion sites and dressings in six nursing homes in Orange County, California, from September 2015 to December 2018. During the latter half of that study (January 2017–December 2018), efforts were made to train on evaluation of insertion sites and to ensure proper dressing changes, but timely catheter removal was not addressed. We identified the subset of the SAFER Lines cohort whose indication for a venous catheter was antibiotic therapy and whose antibiotic end date was known.

For each member of the antibiotic cohort, we collected the following information: nursing home of residence, age, sex, admission date, discharge date (if applicable), prescribed antibiotics, intravenous antibiotic start and end dates, and date of catheter removal. Comorbidities were defined using Elixhauser Comorbidity Conditions based on ICD-10-CM as available in Centers for Medicare and Medicaid Minimum Dataset; state hospitalization data were used to identify infection-related hospitalization and emergency department visits.^4,5^

Residents with a catheter removal date ≥ 1 calendar days after conclusion of intravenous antibiotics were considered to have a catheter that was not removed on time. Time (days) from intravenous antibiotic completion to catheter removal was calculated. Number of catheters removed were categorized by number of days in place beyond antibiotic end and graphically displayed. Catheters without an available removal date were not included in calculations for catheter insertion time after antibiotics were stopped. Analyses were performed using SAS version 9.4 software (SAS Institute, Cary, NC).

## Results

The overall SAFER Lines cohort included 720 residents in six nursing homes. Of these, 193 residents from five nursing homes had 210 had venous catheter access for the sole purpose of intravenous antibiotic therapy with clearly documented completion dates during the study. Of these, 185 (88.1%) were central lines (177 PICCs, 5 subclavian, 2 internal jugular, and 1 femoral) and 25 were midlines. Catheters with missing antibiotic end dates were excluded (n = 22).

**Figure 1.**
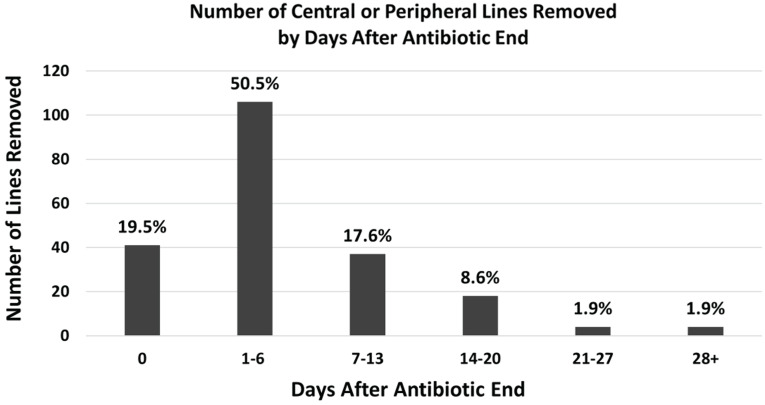
Number of central or peripheral lines removed by days after antibiotic completion. Number of central (n = 185) and peripheral (n = 25) lines removed, categorized by days after antibiotic end date. Percentages calculated among total 210 venous catheters.

Table 1 summarizes nursing home resident and catheter characteristics. Mean (SD) age was 58 (17), 96 (49.7) were male, and mean (SD) comorbidity score was 5.3 (5.4). Among 210 catheters, mean (SD) dwell time was 27.6 (19.6) days and duration of antibiotic treatment was 19.2 (15.7) days. Antibiotic indication was documented for 54 (25.7%) catheters and included skin/soft tissue infection (n = 17, 31.5%), osteomyelitis (n = 16, 29.6%), urinary tract infection (n = 8, 14.8%), pneumonia (n = 7, 13.0%), and the remainder a mixture of other indications (n = 8, 14.8%). Timely removal on the day of antibiotic end occurred for 41 (19.5%) catheters. Of the remaining 169 (80.5%) lines that had late removal,106 (50.5%) were left in place for up to one week and 30% (63/210) remained in place longer than a week, with several lines in place up to one month without documented indication (Figure 1). Mean (SD) excess dwell time across the cohort was 6.8 (7.2) days (Table 1), ranging from 5.2 (6.7) to 8.6 (9.1) days across the five nursing homes. Among the 169 catheters with late removal, 59 (34.9%) had an infection-related hospitalization or emergency department visit of which 36 (61.0%) were in place ≥7 days of antibiotic end.

**Table 1.** Characteristics of nursing home residents with central and peripheral lines receiving intravenous antibiotics

Cohort characteristics	N (%)
Nursing home residents^a^	
Number of residents with lines for antibiotic therapy (N)	193
Age in years (mean, SD)	68 (14.9)
Male sex	96 (49.7)
Comorbid conditions^b^	48 (22.2)
Diabetes mellitus	113 (58.5)
Hypertension	108 (56.0)
Neurological disorders, paralysis, or dementia	66 (34.2)
Renal disease	62 (32.1)
Chronic pulmonary disease	46 (23.8)
Malignant tumor	35 (18.1)
Liver disease	31 (16.1)
Mean (SD) comorbidity score	5.3 (5.4)
Central or peripheral line characteristics	
Total number of lines	210
Line dwell time, mean (SD) days	27.6 (19.6)
Antibiotic duration, mean (SD) days	19.2 (15.7)
Line removed on time^c^	41 (19.5)
Lines remaining in place after antibiotic end	169 (80.5)
Excess days lines in place after antibiotic end	
Mean (SD)	6.8 (7.2)
Median (IQR)	11 (6–25)

a Patients with peripherally inserted central catheter (PICC) line in place in whom indication for a central line was antibiotic therapy and whose antibiotic end date was known.

b Elixhauser Comorbidity Conditions based on ICD-10-CM. (n.d.). https://hcup-us.ahrq.gov/toolssoftware/comorbidityicd10/comorbidity_icd10.jsp.

c Removed on the day that antibiotic treatment ended.

## Discussion

In this study, 80% of nursing homes residents who required central or peripheral venous catheters for intravenous antibiotics did not have their catheters removed on the calendar day antibiotics concluded, with nearly one-third of residents not having it removed until a week or more after antibiotics concluded. Across all residents, failure to remove these venous catheters resulted in an average of 6.8 days of excess dwell time. Each day a catheter is retained without a clear indication for its use poses unnecessary risks for residents, including thrombotic complications, local infection, and central line associated bloodstream infections.^6^

Nursing homes provide complex medical care but often lack the necessary staffing or resources to maintain proactive infection prevention processes.^4^ Resources should be allocated to encourage daily monitoring of line necessity to ensure prompt removal of catheters.^7^ Unfortunately, high-fidelity mechanisms that ensure on-time catheter removal have yet to be implemented in most nursing homes.^8^ The absence of such processes warrants concerted efforts to provide staff training and to set standards of practice to ensure that catheter removal is scheduled when intravenous therapy is discontinued. Although physicians are generally present in nursing homes only once or twice per week, scheduled antibiotic administration and known stop days should allow timely removal of catheters by nurses who are present at all shifts even when physicians may not be on-site.^9^

Timely catheter removal should be included in structured training of nurses and physicians related to catheter placement, maintenance, and discontinuation. With increasing use of electronic medical records in nursing homes, it is possible to implement electronic prompts tied to an antibiotic order which could prespecify the end date of intravenous therapy.^10^ This would tether catheter removal to the completion of therapy and ensure accountability among care staff.

This study has several limitations. The sample size is limited to five nursing homes in a single geographic region which may limit generalizability. In addition, this study was retrospective and reasons for delayed removal were not assessed. Similarly, any harm from delayed removal was not collected or quantified.

In summary, central and peripheral venous catheters used for antibiotic therapy in nursing homes are more often than not overdue for removal. Protocols are urgently needed to ensure that removal dates are linked to antibiotic completion dates to reduce the risk of thrombotic and infectious complications.
